# *Glossina* spp. gut bacterial flora and their putative role in fly-hosted trypanosome development

**DOI:** 10.3389/fcimb.2013.00034

**Published:** 2013-07-24

**Authors:** Anne Geiger, Marie-Laure Fardeau, Flobert Njiokou, Bernard Ollivier

**Affiliations:** ^1^UMR 177 InterTryp, IRD-CIRADMontpellier, France; ^2^Université Aix-Marseille, Université du Sud Toulon-Var, CNRS/INSU, IRD, MIO, UM 110Marseille, France; ^3^Department of Science, University of Yaoundé IYaoundé, Cameroon

**Keywords:** human African trypanosomiasis, bacteriome, trypanosome, tsetse flies, interactions

## Abstract

Human African trypanosomiasis (HAT) is caused by trypanosomes transmitted to humans by the tsetse fly, in which they accomplish their development into their infective metacyclic form. The crucial step in parasite survival occurs when it invades the fly midgut. Insect digestive enzymes and immune defenses may be involved in the modulation of the fly's vector competence, together with bacteria that could be present in the fly's midgut. In fact, in addition to the three bacterial symbionts that have previously been characterized, tsetse flies may harbor additional bacterial inhabitants. This review focuses on the diversity of the bacterial flora in *Glossina*, with regards to the fly species and their geographical distribution. The rationale was (i) that these newly identified bacteria, associated with tsetse flies, may contribute to vector competence as was shown in other insects and (ii) that differences may exist according to fly species and geographic area. A more complete knowledge of the bacterial microbiota of the tsetse fly and the role these bacteria play in tsetse biology may lead to novel ways of investigation in view of developing alternative anti-vector strategies for fighting human—and possibly animal—trypanosomiasis.

## Introduction

A comprehensive understanding of the biology of insects requires investigations on the microbial content of their guts (Steinhaus, 1960). Insects are hosts for a large panel of microorganisms that have developed a variety of interactions ranging from mutualistic to parasitic (Jeyaprakash et al., 2003; Schmitt-Wagner et al., 2003; Campbell et al., 2004; Hongoh et al., 2005). Some of these interactions have been quite well characterized, owing to their ecologic and/or economic importance. However, the exact nature of many of these interactions remains poorly understood and poorly documented.

Human African trypanosomiasis (HAT), or sleeping sickness, caused by trypanosomes transmitted to humans by the tsetse fly (*Glossina* spp.), belongs to the neglected tropical diseases affecting more than 1 billion people worldwide (Fèvre et al., 2008; Welburn et al., 2009). Regarding sleeping sickness itself, 60 million people are living in HAT-risk areas in the 36 countries that are listed by WHO as being endemic for the disease, among which only 10–15% really undergo epidemiological control (Cattand et al., 2001). This means that the actual number of HAT cases is probably much higher than reported and that HAT remains a serious public health problem even though the prevalence of HAT now seems to be decreasing (Barrett, 2006; WHO, 2010; Simarro et al., 2011). Unless treated the disease is fatal. The drugs currently used to fight the disease are not satisfactory, some are toxic, and all are difficult to administer (Barrett, 2006). Furthermore, trypanosome resistance to some drugs has developed and is increasing (de Koning, 2001). Therefore new strategies to combat the disease need to be developed.

To be transmitted to the mammalian host, trypanosomes must first establish in the insect midgut and, upon their migration to the salivary glands, they have to undergo a maturation process. When the fly feeds on infected mammalian hosts, trypanosomes enter the fly midgut, where they rapidly differentiate into procyclic forms. Then they either die in the midgut of refractory individuals or survive to yield persistent procyclic infections in susceptible insects. Once established, parasites migrate toward the salivary glands where they differentiate into epimastigote forms and, finally, into infectious metacyclic forms (maturation step) that can be transmitted to naïve mammals by the fly when taking another blood meal (Vickerman et al., 1988; Van Den Abbeele et al., 1999). The factors involved in the establishment step are still largely unknown. However, several factors are believed to be involved in this step among which the fly's digestive enzymes and immune defenses and the intestinal microbial flora (Welburn and Maudlin, 1999; MacLeod et al., 2007; Wang et al., 2009, 2012; Weiss and Aksoy, 2011). As reviewed by Dillon and Dillon (2004), insects harbor, mainly in the intestinal organs, diverse communities of microorganisms. The tsetse fly harbors three symbiotic microorganisms (Aksoy, 2000): (i) the obligate primary symbiont, *Wigglesworthia glossinidia* (Aksoy, 2000), which synthesizes B vitamins (Akman et al., 2002) that the fly is unable to synthesize and which are absent from its blood diet; (ii) *Wolbachia* (O'Neill et al., 1993), belonging to the *Rickettsiaceae* family, which infects a broad range of insect species, causing a variety of reproductive abnormalities, and cytoplasmic incompatibility in tsetse flies (Alam et al., 2011); and (iii) *Sodalis glossinidius*, belonging to the *Enterobacteriaceae* family, which has been shown to be involved in the fly's vector competence (Dale and Maudlin, 1999). Although most of the studies dedicated to insect gut microbiota focused on the contribution of microbial endosymbionts to the host's nutritional homeostasis (Dillon and Dillon, 2004), others examined the role of gut bacteria in preventing pathogen development (Pumpuni et al., 1993, 1996; Welburn and Maudlin, 1999; Gonzalez-Ceron et al., 2003; Azambuja et al., 2004). Since the trypanosomes have to complete part of their lifecycle within their vector, particularly in its gut, the concomitant presence of diverse bacteria, if any, could affect the parasite's lifecycle and finally the fly's vector competence. Therefore, our knowledge on the composition of the tsetse fly midgut bacterial flora must be improved to gain more detailed insight into the potential interactions between these bacteria and the insect harboring *Trypanosoma*, and/or even with the parasite itself.

This article reviews the present knowledge on the fly's gut-associated bacteria, other than symbionts, and suggests novel ways of investigation.

## Diversity of microbiota in tsetse flies

**Figure 1:**
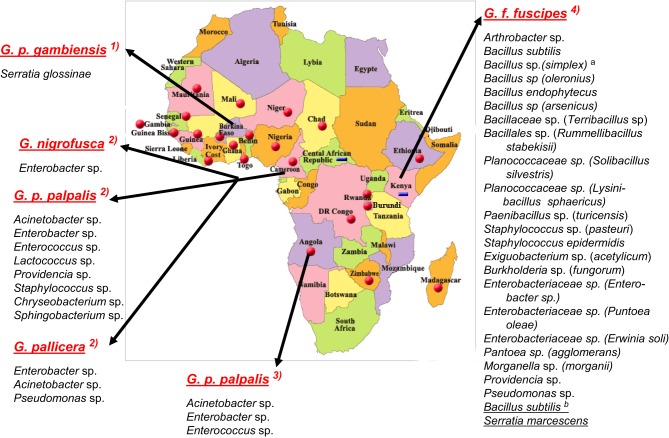
**Bacterial species characterized in the midgut of tsetse flies species from sub-Saharan African countries.** According, respectively, to: (1) Geiger et al. (2010); (2) Geiger et al. (2011); (3) Geiger et al. (2009); (4) Lindh and Lehane (2011). Bacteria from *G. f. fuscipes*: ^*a*^the species between brackets are the closest relatives according to RDPII (Maidak et al., 2001); ^*b*^the species underlined were identified with culture-independent methods. No bacteria were identified in *G. caliginea*.

While the bacterial flora composition of a few of insects [*Drosophila* and several mosquitoes (Pumpuni et al., 1993, 1996; Broderick and Lemaitre, 2012)] has been investigated for years and is fairly well documented, the bacterial flora composition of the tsetse fly has only recently gained attention. Studies on tsetse flies have been conducted on insectary-reared *Glossina palpalis gambiensis* flies and on flies belonging to several *Glossina* species collected in HAT foci in two Africa countries—Angola and Cameroon (Geiger et al., 2009, 2010, 2011)—and on G. *fuscipes fuscipes* flies from Kenya (Lindh and Lehane, 2011) (Figure 1). It is noteworthy that, using a culture-dependent isolation method and a similar enrichment procedure throughout the studies, the former group evidenced differences in the bacterial flora composition not only with respect to the fly species, but also to their geographical origin. The approach used included dilution series (which ranged from 10^−6^ to 10^−10^, depending on the study) of the midgut before bacterial enrichment, in order to ensure the isolation of microorganisms that have actively multiplied in the gut and that can therefore be considered as true gut inhabitants; this process rules out bacteria that are merely transient residents. The isolated bacteria were then identified using molecular phylogeny identification. However, this culture-dependent method does not allow the identification of non-cultivable bacteria. In contrast, the group (Lindh and Lehane, 2011) working on flies collected in East Africa used both culture-dependent and culture-independent approaches that are expected to allow the characterization of not easily cultivated—or even non-cultivable—bacteria, but possibly also of bacteria that are simply in transit in the flies' gut.

### The bacterial flora of tsetse flies from angola, cameroon, and kenya

**Figure 2:**
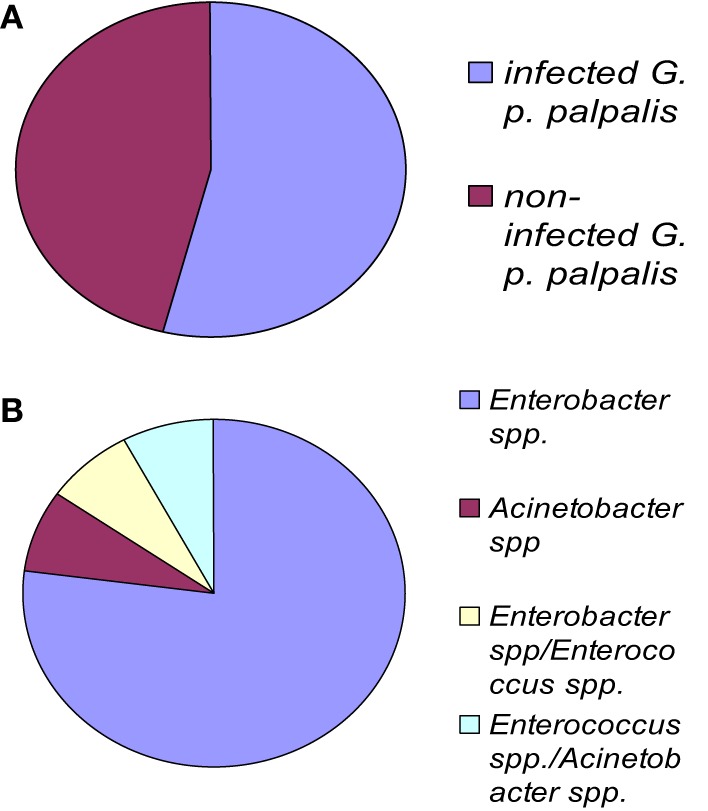
***G. palpalis palpalis* infections in the Maria Teresa focus in Angola according to Geiger et al. (2009). (A)** Prevalence of infection in tsetse flies; **(B)** frequency of occurrence for each infection type. “Multiple” names indicate the occurrence of a mixed infection.

**Figure 3:**
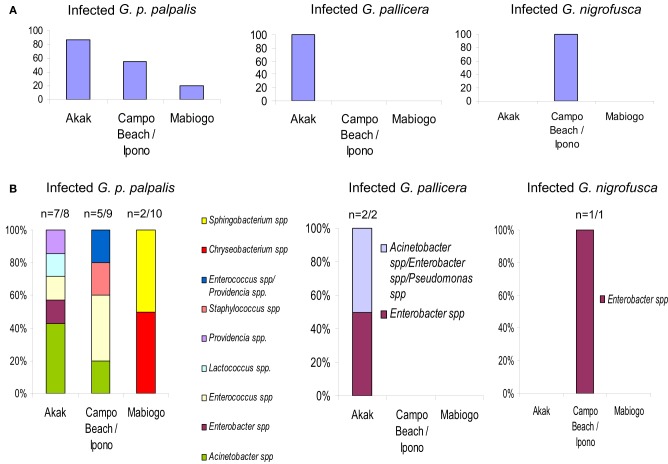
***G. palpalis palpalis*, *G. pallicera* and *G. nigrofusca* fly' bacterial infections occurring in the different villages (Akak, Campo Beach/Ipono and Mabiogo) of the Campo focus in Cameroon, according to Geiger et al. (2011). (A)** Prevalence of bacterial infection in the different tsetse fly species; **(B)** bacterial infection frequency per fly species and per village. “n” indicates the number of bacterial infected flies vs. the number of collected flies. “Multiple” names indicate the occurrence of a mixed infection.

The fly species collected and studied differed from one country to another: *Glossina palpalis palpalis* in Angola, *G. p. palpalis*, *G. pallicera*, *G. nigrofusca*, and *G. caliginea* in Cameroon, and *G. fuscipes fuscipes* in Kenya, which allows limited comparisons only between fly species from different countries (Figure 1). However, one may note the overall relatively high fly infection rates by bacteria for all three countries: 54% in Angola, 53% in Cameroon (Figures 2A and 3A), and 72% in Kenya (42% when discarding the bacteria isolated from the outer cuticle of the mosquitoes), despite the differences observed in the fly species. Similarly, the prevalence of Gram-negative bacteria was much higher than the Gram-positive bacteria. Finally, most often an individual fly harbored only one bacterial species; mixed infections were sometimes observed whatever the fly species studied (Figures 2B and 3B). However, the number of bacterial isolates characterized, three per fly, was low and therefore the prevalence of mixed infection could be underestimated.

The overall high diversity of bacterial species was also unexpected (Figure 1) with respect to (i) the geographic origin of the flies: 3 bacterial species in flies from Angola, 9 in Cameroon (Figures 2, 3), 22 in Kenya (+2 identified by the culture-independent method), and/or (ii) the fly species: 22 (+2 by molecular approaches) in *G. fuscipes fuscipes*, 8 in *G. p. palpalis*, 3 in *G. pallicera*, 1 in *G. nigrofusca*, none in *G. caliginea*. The number of *G. pallicera*, *G. nigrofusca*, and *G. caliginea* flies collected and analyzed was very low making conclusions about the number and types of bacterial species in these flies limited at this time.

Besides these similarities, substantial differences are noted when comparing the results recorded for different countries; in fact, the overall bacterial species are assigned to four different phyla in which they are nevertheless unevenly distributed with reference to the geographic origin of the flies: Actinobacteria, 4% in Kenya, 0% in Angola and Cameroon; Proteobacteria: 36% in Kenya, 66% in Angola, and 44% in Cameroon; Firmicutes: 60% in Kenya, 33% in Angola and Cameroon, and Bacteroidetes: 0% in Kenya and Angola, 22% in Cameroon. In addition, when comparing the overall bacteria species identified in the two West African countries (on *G. p. palpalis*, *G. pallicera*, and *G. nigrofusca*) with those characterized in Kenya (on *G. f. fuscipes*), only four species were found to be common: *Enterobacter* spp., *Providencia* spp., *Pseudomonas* spp., and *Staphylococcus* spp. (Figure 1). However, differences in bacterial culture conditions (as opposed to differences in geographic origin) may account for differences in bacterial species. Finally, while a large diversity of bacteria was found in field-collected tsetse flies, only one bacterial species, a novel one pertaining to the *Serratia* genus, *S. glossinae*, was isolated from insectary-reared fly midguts of *G. p. gambiensis*, trapped several years before in Burkina Faso (Geiger et al., 2010) (Figure 1).

### Differences in the bacterial diversity in tsetse flies collected in three areas belonging to the same HAT focus

In contrast to the substantial differences in the diversity of bacterial gut inhabitants recorded according to the geographic origin of the flies, it could be expected that such differences would be much more limited in flies collected in a restricted area. This was not the case, as shown by the results of an investigation carried out in three villages (Akak, Campo Beach/Ipono and Mabiogo) located into the same HAT focus, Campo, in southern Cameroon.

The flies sampled in Campo belonged to four *Glossina* species, *G. p. palpalis*, *G. pallicera*, *G. caliginea*, and *G. nigrofusca; G. p. palpalis* accounted for 94% of the fly community. Nine bacterial species were isolated and identified from these sampled flies: *Acinetobacter* spp., *Enterobacter* spp., *Providencia* spp., *Pseudomonas* spp., *Enterococcus* spp., *Lactococcus* spp., and *Staphylococcus* spp., *Chryseobacterium* spp., and *Sphingobacterium* spp.; except for *Pseudomonas* spp., all of them could be isolated from *G. p. palpalis* flies (Figures 1, 3).

The large differences in fly infection rates recorded with reference to the collecting sites were surprising. In the most representative species, *G. p. palpalis*, 87.5% of the flies collected in Akak were infected, in contrast to 55.5% of the flies from Campo Beach/Ipono, and only 20% of those from Mabiogo (Figure 3A). Furthermore, considering *G. p. palpalis*, the distribution of the different bacteria identified was also very uneven with respect to the origin of the flies. In Mabiogo, the infection rate was the lowest. Two bacterial species were identified: *Chryseobacterium* spp. and *Sphingobacterium* spp. These bacteria were not identified in the flies sampled in the two other villages in the performed surveys. Similarly, *Enterobacter* and *Lactococcus* spp. infections were restricted to flies collected in Akak (Geiger et al., 2011), and finally, four bacteria species were isolated from flies from Campo Beach/Ipono (*Acinetobacter* spp., *Providencia* spp., *Enterococcus* spp., and *Staphylococcus* spp.) (Figure 3B). However, since these surveys looked at three bacterial isolates per fly, it is possible that the prevalence of each bacterial species could be underestimated in the different villages tested.

### Origin of the gut bacteria and their diversity according to the fly species and their geographic location

The high prevalence and diversity of bacteria in tsetse flies is unexpected given that these flies are monophagous as they only feed on vertebrate blood throughout their life span. In wild populations of mosquitoes, the origin of the midgut bacteria is unknown (Pumpuni et al., 1996; Straif et al., 1998), as in tsetse flies. However, differences in the environmental conditions and in the food supply may influence the diversity of the bacterial communities harbored. This hypothesis could be acceptable if one considers that the fly may swallow bacteria present in the environment, particularly on the skin of the animals on which it feeds. This possibility cannot be excluded since Poinar et al. (1979) demonstrated that, when applied to the ears of rabbits used as tsetse fly-feeding hosts, the bacterium *S. marcescens* was ingested during the blood meal and multiplied in the fly's gut. Tsetse flies were shown to feed on a variety of hosts (Simo et al., 2008; Farikou et al., 2010), which probably carry diverse bacteria on their hair and skin, thus implying the possibility of the flies being infected by these bacteria. Nevertheless, the mechanism may be more complex since the *G. p. palpalis* flies collected in the three villages of the Campo HAT focus differed in their bacterial inhabitants, even though they developed in similar environmental conditions.

## Involvement of midgut bacteria in the insect vector competence and its survival

While investigations on the potential effect of gut microbiota on tsetse fly vector competence are nearly non-existent, such studies have been successfully conducted on other insects.

Gonzalez-Ceron et al. (2003) reported that the *Plasmodium vivax* sporogonic development in field-collected *Anopheles albimanus* was blocked by bacteria inhabiting the mosquitoes' midgut (Gonzalez-Ceron et al., 2003). When feeding laboratory-reared adult anopheline species with either Gram-negative or Gram-positive bacteria together with *Plasmodium falciparum* gametocytes, it was shown that Gram-negative, but not Gram-positive, bacteria partially or totally inhibited the formation of oocysts (Pumpuni et al., 1993, 1996). In contrast, working on field-collected mosquitoes, Straif et al. (1998) showed that the presence of Gram-negative bacteria in the midgut did not influence the number of *Anopheles funestus* infected with *P. falciparum* sporozoites, while Gram-positive bacteria significantly enhanced the incidence of mosquitoes that contained sporozoites. Furthermore, feeding mosquitoes with gentamicin significantly increased the number of *Plasmodium*-infected mosquitoes (Beier et al., 1994). In *Anopheles albimanus*, co-infections with *S. marcescens* and *Plasmodium vivax* resulted in only 1% of mosquitoes being infected with parasites, compared to a 71% infection rate in control mosquitoes (Gonzalez-Ceron et al., 2003). Recently, a significant positive correlation was observed between *P. falciparum* infection and the presence of *Enterobacteriaceae* in the mosquitoes' midgut (Boissière et al., 2012). In sandflies (*Phlebotomus papatasi*), microbial infections significantly reduced the rates of infection with *Leishmania major* (Schlein et al., 1985). In addition, strains of *Pseudomonas fluorescens* (Mercado and Colon-Whitt, 1982), as well as of *S. marcescens* (which was isolated from *Rhodnius prolixus*) (Azambuja et al., 2004) have been reported to be able to lyse *Trypanosoma cruzi in vitro*. All these examples show potential implication of bacteria isolated from insects in their vector competence.

Some of the bacterial genera/species that were found in at least one species of tsetse fly (Geiger et al., 2009, 2011) have been shown to affect other insects. *Stomoxys calcitrans* fly larvae require the presence of *Acinetobacter* spp. for complete development (Lysyk et al., 1999). Conversely, several other bacterial species including *Providencia* spp. and *Pseudomonas* spp. are close relatives of known insect bacteria (Jackson et al., 1995; Lacey, 1997). In addition, a number of Gram-negative and Gram-positive bacteria such as *S. marcescens*, *Providencia rettgeri*, and several *Bacillus* spp. induce mortality in *G. m. morsitans* (Kaaya and Darji, 1989). Furthermore, *S. marcescens* has been shown to cause increased mortality in *Anopheles albimanus* mosquitoes and in *G. pallidipes* flies (Poinar et al., 1979; Gonzalez-Ceron et al., 2003). Other bacteria isolated from field tsetse flies (Geiger et al., 2009, 2011; Lindh and Lehane, 2011) were assigned to the genus *Lactobacillus*, some members of which are reported to be pathogenic to plants and animals whereas other *Lactobacilli* are commonly found as members of human microbiota (Hammes and Hertel, 2006).

Symbionts have also been implicated in vector competence and/or tsetse fly survival. Studies from Weiss et al. (2011, 2012) have shown that *Wigglesworthia* protect against *Escherichia coli* infection and promote tsetse immune system development. Moreover, interactions between *Wigglesworthia* and the tsetse peptidoglycan recognition protein (PGRP-LB) may be involved in trypanosome transmission (Wang et al., 2009). Weiss et al. (2013) showed that trypanosome infection in the tsetse fly gut was influenced by microbiota-regulated host immune barriers. Geiger et al. (2007) showed an association between the presence of specific genotypes of *Sodalis* and *G. p. gambiensis* midgut infection by *Trypanosoma brucei gambiense* or *Trypanosoma brucei brucei*.

## Mechanisms potentially involved in the modulation of parasite infection by midgut microbiota

**Figure 4:**
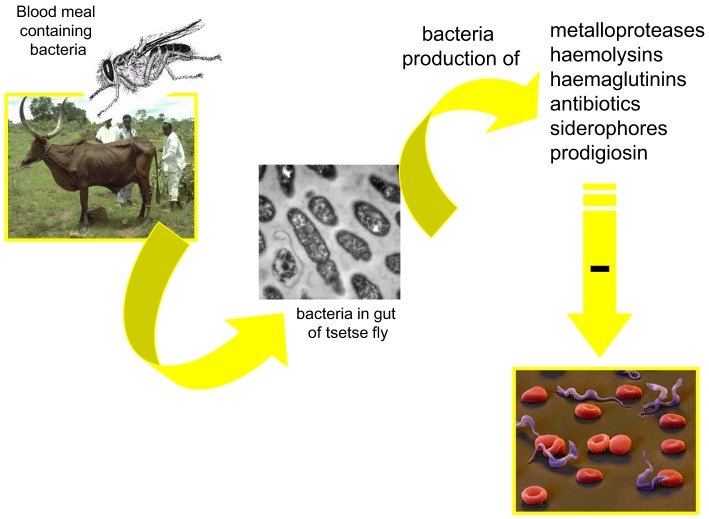
**Possible mechanism involved in the modulation, by the midgut microbiota, of the tsetse fly infection by trypanosomes**.

Several mechanisms may be involved in the modulation of parasite infection by midgut microbiota. One could be the competition for limited resources or the production of antiparasitic molecules by the bacteria inhabiting the vectors' gut. Toxic molecules (Figure 4) with potential antiparasitic activity have been identified. Among them are cytotoxic metalloproteases produced, for example, by *S. marcescens* and *Pseudomonas aeruginosa* (Maeda and Morihara, 1995) or hemolysins secreted by *Enterobacter* spp., *E. coli*, *S. marcescens*, and *Enterococcus* spp. (Hertle et al., 1999; Coburn and Gilmore, 2003). Antibiotics can be produced by *Serratia* spp.(Thomson et al., 2000); hemagglutinins (Gilboa-Garber, 1972) and siderophore by *P. aeruginosa* (Schalk et al., 2002). An antitrypanosomal factor has been shown to be produced by *P. fluorescens* (Mercado and Colon-Whitt, 1982). Pigments such as prodigiosin are produced by the Gram-negative bacteria such as *Serratia* spp. and *Enterobacter* spp. (Moss, 2002). They induce the fragmentation of DNA, characterizing an apoptotic action of the toxin (Díaz-Ruiz et al., 2001; Montaner and Perez-Tomas, 2003). Prodigiosin was shown to be toxic for *P. falciparum* (Lazaro et al., 2002) and *T. cruzi* (Azambuja et al., 2004). Free hemoglobin, resulting from the hemolysis of the blood meal in the digestive tract of vector insects (Azambuja et al., 2004) has been suggested to be a ready source of iron for bacteria and would contribute to the massive increase in the gut bacteria population, following feeding. However, toxic molecules have not been shown to be constitutively expressed and the production of these may even be indirectly correlated with bacterial density.

Dong et al. (2009) suggested the bacteria-mediated anti-plasmodium effect was due to the mosquito's antimicrobial immune responses, possibly through the activation of basal immunity. Recently, in Zambia, *Enterobacter* spp. were isolated from wild mosquitoes resistant to infection with *P. falciparum*. It was suggested the anti-*Plasmodium* effect was caused by bacterial generation of reactive oxygen species (Cirimotich et al., 2011).

## Perspectives

It is crucial to investigate whether any of the recently identified bacteria in tsetse could modulate the fly vector's competence, as do the flies' endosymbionts (Welburn and Maudlin, 1999), and as has already been reported in other insect parasite vectors (Pumpuni et al., 1993, 1996; Straif et al., 1998; Gonzalez-Ceron et al., 2003). Such modulation may occur through direct inhibitory bioactivity, by secreted enzymes or toxins focused on the parasitic trypanosomes. Alternatively, microbiota may constrain pathogen development indirectly by activating or enhancing the host immune system that in turn could clear the parasite; this effect was previously reported for *Wigglesworthia* affecting PGRP-LB (Wang et al., 2009; Weiss et al., 2013). Investigations on several insect systems indicate that both direct and indirect microbiota-induced phenotypes occur (Dong et al., 2009; Cirimotich et al., 2011). Finally, understanding the mechanisms governing the association between of tsetse flies and the hosted bacteria, and determining how the association is controlled, are important issues. These issues could be addressed by monitoring the diversity and density of bacteria in flies throughout their life cycle and by investigating the possible transmission of these bacteria species by the female fly to its progeny, as occurs for the maternal transmission of the three *Glossina* endosymbionts.

In wild populations, differences in environmental conditions and in food supply may influence the diversity of the bacterial communities harbored by the flies. This could explain the diversity in the flies' gut bacterial inhabitants and in fly infection rates reported in tsetse fly communities from Angola, Cameroon and Kenya, and therefore point out the need to multiply and diversify the fly collecting areas. Moreover, a greater number of samples has to be collected in order to better assess the occurrence of co-infections and to evidence the possible involvement of the gut-hosted bacteria in the tsetse fly.

All these investigations deserve to be undertaken as they may open novel avenues for tsetse vector competence control through manipulation of gut microbial communities, which in turn may result in novel HAT control strategies.

### Conflict of interest statement

The authors declare that the research was conducted in the absence of any commercial or financial relationships that could be construed as a potential conflict of interest.
